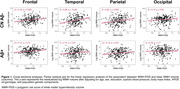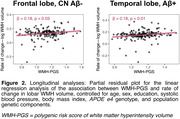# Polygenic predisposition to white matter hyperintensities does not contribute to amyloid or tau progression in Alzheimer’s disease

**DOI:** 10.1002/alz.090657

**Published:** 2025-01-09

**Authors:** Lukai Zheng, Simon Frerich, Nicolai Franzmeier, Rainer Malik, Michael Ewers, Alzheimer's Disease Neuroimaging Initiative

**Affiliations:** ^1^ Institute for Stroke and Dementia Research (ISD), University Hospital, LMU, Munich, Bayern Germany; ^2^ Institute for Stroke and Dementia Research (ISD), University Hospital, LMU, Munich Germany

## Abstract

**Background:**

White matter hyperintensities (WMH) are prevalent Alzheimer’s disease (AD). However, the role of WMH in the etiology of AD is debated. Specifically, a key question is whether higher WMH is predictive of higher beta‐amyloid (Aβ) and fibrillar tau accumulation. Here, we assessed whether a genetic predisposition to higher WMH (assessed via polygenic score [PGS] of WMH based on a large‐scale population‐based GWAS) is associated with higher rates of WMH volume and amyloid‐ and tau‐PET accumulation in elderly individuals.

**Methods:**

We included 338 participants within the AD spectrum defined by amyloid‐PET positivity, encompassing 135 cognitively normal (CN Aβ+), 122 mild cognitive impairment (MCI Aβ+), 81 AD dementia, and in addition 287 amyloid‐negative controls (CN Aβ‐) from from ADNI. Flortaucipir‐PET was available in a subset of 92 Aβ+ & 85 CN Aβ‐ participants. WMH were segmented via a deep learning‐based algorithm on FLAIR images and partitioned into 5 lobar volumes. Based on GWAS in 35,000 individuals from the UKBiobank with global WMH volume as the dependent variable, we generated a PGS including 20 SNPs (p<5×10^−8^). Centiloids of global cortical amyloid‐PET and temporal‐meta ROI values of tau‐PET SUVR were computed. All linear regression analyses were stratified by group (CN Aβ‐ vs pooled Aβ+) and controlled for age, sex, education, systolic blood pressure, BMI, APOE e4 genotype, and population genetic components.

**Results:**

A higher PGS was associated with higher cross‐sectional WMH volume in the frontal, parietal, and temporal lobes in both the CN Aβ‐ and the whole Aβ+ group (except for temporal WMH; Figure 1). Longitudinally, higher PGS was associated with faster increases in frontal WMH volume in CN Aβ‐ (β=0.18, p=.03) and in the temporal lobe in the Aβ+ group (β=0.18, p=.01, Figure 2) over 2.8 years on average. Conversely, neither PGS nor baseline WMH volume (or modifiable risk factors) predicted increase in amyloid‐ or tau‐PET (mean follow‐ups: 3.9 & 2.8 years).

**Conclusion:**

Genetic predisposition to WMH was associated with higher WMH volume in a region‐dependent manner, but the increases in WMH were associated neither with Aβ‐ nor tau‐PET accumulation, suggesting that white matter lesions develop independently from AD pathologies.